# Testing a Simulation Model for the Response of Tomato Fruit Quality Formation to Temperature and Light in Solar Greenhouses

**DOI:** 10.3390/plants13121662

**Published:** 2024-06-15

**Authors:** Yongdong Qin, Ao Gong, Xigang Liu, Nan Li, Tuo Ji, Jing Li, Fengjuan Yang

**Affiliations:** 1 College of Horticulture Science and Engineering, Shandong Agricultural University, Tai’an 271018, China; 2 College of Information Science and Engineering, Shandong Agricultural University, Tai’an 271018, China; 3 Key Laboratory of Biology and Genetic Improvement of Horticultural Crop (Huang-Huai Region), Ministry of Agriculture and Rural Affairs, Tai’an 271018, China; 4 Shandong Collaborative Innovation Center for Fruit and Vegetable Production with High Quality and Efficiency, Tai’an 271018, China

**Keywords:** tomato, fruit quality, light and temperature, simulation model, TEP

## Abstract

Temperature and light are the key factors affecting the formation of tomato fruit quality in greenhouse cultivation. However, there are few simulation models that examine the relationship between tomato fruit quality formation and temperature and light. In this study, a model was established that investigated the relationships between soluble sugar (SSC), organic acid content (OAC), and SSC/OAC and the cumulative product of thermal effectiveness and photosynthetically active radiation (TEP) during the fruit-ripening period in a solar greenhouse. The root mean square error (RMSE) values were calculated to compare the consistency between the simulated and measured values, and the RMSE values for SSC, OAC, and SSC/OAC were 0.09%, 0.14%, and 0.358, respectively. The combined weights of quality indicators were obtained using the analytic hierarchy process (AHP) and entropy weighting method, ranking as SSC > OAC > SSC/OAC > CI > lycopene > Vc > fruit firmness. The comprehensive fruit quality evaluation value was obtained using the TOPSIS method (Technique for Order Preference by Similarity to an Ideal Solution) and a simulation model between comprehensive tomato fruit quality and TEP was explored. This study could accurately simulate and quantify the accumulation of tomato fruit quality during fruit ripening in response to environmental conditions in a solar greenhouse.

## 1. Introduction

Tomatoes are one of the most grown vegetables in the world, with a global annual output of 170 million t. As one of the main production areas, China’s tomato-planting area was 1.109 million hm^2^ in 2018, with a yield of 64.832 million t [1,2]. With the abundant supply of vegetables, consumers increasingly demand high-quality vegetables. Tomatoes have become one of the most popular vegetables because of their rich minerals, vitamins, organic acids, essential amino acids, and other nutrients [3,4].

Environmental factors in the greenhouse are the key factors affecting the growth and development of tomatoes. In recent years, with the global warming, extreme weather events frequently occur, which have a serious impact on the growth, development, yield, and quality of crops [5,6]. Temperature is one of the key environmental factors affecting crop yield and quality; different crops have their optimum temperature ranges. High temperatures cause accelerated ripening of tomatoes, so average harvested fruit weights are often reduced [7,8]. Yield and quality are also affected by low temperatures and the fruit-ripening time is prolonged when plants are grown at lower temperatures [9,10]. Changes in daily temperature patterns may affect the growth and metabolism of plants and fruit, and ultimately affect fruit quality [11,12]. At high temperatures (30–35 °C), SSC increases, while OAC decreases [13]. The biosynthesis of lycopene is strongly inhibited at temperatures below 12 °C, while temperatures above 32 °C hinder this process and lead to a decrease in its content [14,15], reaching a maximum concentration at 25 °C [16]. Studies have shown that the vitamin C concentration is seen to increase when plants are high-temperature-stressed at the flowering stage [17]. Light is the driving force of plant photosynthesis, and the accumulation of photosynthetic assimilates is crucial to yield [18]. In addition, light can affect plant transpiration by regulating the closure of leaf stomata, thus influencing crop transport and growth [19,20]. A prolonged light treatment increases the starch and sugar contents in tomato fruit, and promotes the synthesis of anthocyanins in apple fruit skin and the accumulation of soluble sugars in apple flesh [21,22]. Extending the light duration not only promotes fruit coloration, but also the accumulation of fruit sugar; the appearance quality and fresh eating quality of fruit are significantly improved [23,24]. In the life activities of plants, there is a complex interaction between temperature signals and light signals, and temperature and light together affect the growth and development of plants [25,26,27].

Greenhouse cultivation is one of the main methods for tomato production, but it often faces problems such as high temperatures in summer and low temperatures and light in winter, which seriously affects tomato yield and quality. The interaction of multiple environmental factors affects the growth and development of tomatoes. With accumulating solar radiation (CSR), cumulative heat unit (CHU), and steam pressure deficiency (VPD) as variables, a multi-linear regression (MLR) method was used to establish a model for fertility, flowers, and fruit [28]. Wu et al. (2021) established a relationship model of above-ground biomass based on GDDs (growing degree days) and then analyzed the influence of three irrigation regimes on greenhouse-grown tomatoes [29]. Correlations between tomato seedling quality characteristics (root–shoot ratio, G value, and healthy indices) and TEP (thermal effectiveness and photosynthetically active radiation) have been explored to establish models [30]. Most previous studies have mainly focused on exploring the optimal combination of temperature and light environments at the tomato seedling stage, exploring the relationship between tomato growth and development and environmental factors [31,32]. However, fruit quality is the main source of value of tomatoes and there are few reports on the demand characteristics of environmental factors for fruit quality during the fruit-ripening period.

Tomato fruit quality is a comprehensive concept that is the result of the joint action of various quality indicators [33]. It mainly includes the appearance quality, taste quality, and nutritional quality [34]. Soluble sugar and acid jointly determine the taste quality of tomatoes [35]. Lycopene and Vc are the main nutrients in tomato fruit and have many health benefits. Lycopene represents 80 percent of the carotenoids in tomatoes and Vc has reducing and chelating properties, helping to enhance the body’s absorption of iron [36,37]. The appearance quality of tomato fruit (color, size, etc.) is also an important factor when determining the quality of tomato products. Many methods have been used to evaluate tomato quality such as the analytic hierarchy process (AHP), principal component analysis (PCA), Gray relational analysis (GAR), and entropy method as well as TOPSIS (Technique for Order Preference by Similarity to Ideal Solution) [38,39]. However, some methods are influenced by subjective factors and others are evaluated based on raw data, resulting in inaccurate results. Therefore, a combination of multiple evaluation methods can obtain more accurate evaluation results. Based on GAR and PCA, a comprehensive fruit quality grade was calculated using the combined evaluation method [34]. By combining the weights of AHP and the entropy method to obtain combined weights and using a multi-level fuzzy evaluation to obtain comprehensive evaluation indicators, a multi-factor regulation model of water and fertilizer for the comprehensive growth of cherry tomatoes was constructed [40]. The combined weights were obtained by combining AHP and the entropy method weights using game theory and TOPSIS was used to comprehensively evaluate different experimental treatments. Thus, a multi-factor coupled regulation model was constructed for a greenhouse environment based on the comprehensive growth of cherry-tomato seedlings [32].

In this study, we planted 10 batches of tomatoes throughout the first and second half of the year in a solar greenhouse with natural environmental conditions. The relationship between the appearance quality, taste quality, and nutritional quality of tomato fruit and temperature and light conditions was analyzed. A suitable range of temperature and light conditions for tomato ripening was determined. A model was established that examined the relationships between soluble sugar (SSC), organic acid content (OAC), and SSC/OAC in tomato fruit and the cumulative product of TEP during the fruit-ripening period in a solar greenhouse. A comprehensive evaluation analysis was also performed. Our results uncovered the demand characteristics of temperature and light environments during the fruit-ripening period, providing a theoretical basis for the production of high-quality tomatoes in a solar greenhouse.

## 2. Materials and Methods

### 2.1. Plant Materials and Growth Conditions

Tomato cultivation was conducted in the solar greenhouse of the Science and Technology Innovation Park of Shandong Agricultural University. ‘Kaideyali 1832’, one of the commonly used cultivated tomato varieties in Shandong Province, China, was selected as the experimental material. The planting dates are shown in Table 1. A Yamasaki tomato nutrient solution was selected for fertilizer and water management using a regular drip irrigation of water and fertilizer integrated machine [41].

### 2.2. Measurements

#### 2.2.1. Meteorological Data

Environmental data such as temperature and solar radiation in the solar greenhouse were automatically collected by “Shennong IOT” equipment, developed by the Big Data Center of Shandong Agricultural University. In total, 6 sensors were evenly distributed in the solar greenhouse. Their positions were set at the tomato apexes and the middle of the canopy, and the data were uploaded every 5 min.

Temperature and solar radiation are the two most important factors for tomato fruit development. To consider the combined effect of the two factors, we employed the concept of an accumulated production of photosynthetically active radiation and relative thermal effectiveness (TEP). TEP can be calculated as follows [42].

(1) $$RTE=\left\{\begin{array}{c} \begin{array}{cc} 0 & \left(T<T_{b}\right)\\ \left(T-T_{b}\right)/\left(T_{ab}-T_{b}\right) & \left(T_{b}\leq T<T_{ab}\right) \end{array}\\ \;\\ \begin{array}{cc} 1 & \left(T_{ab}\leq T\leq T_{ou}\right)\\ \left(T_{m}-T\right)/\left(T_{m}-T_{ou}\right) & \left(T_{ou}<T\leq T_{m}\right)\\ 0 & \left(T<T_{m}\right) \end{array} \end{array}\right.$$

(2) $$HTEP=RTE\times PAR\times {3600\times 10}^{-6}$$

(3) DTEP=∑(HTEP)

(4) $${TEP}_{\left(i+1\right)}={TEP}_{\left(i\right)}+D{TEP}_{\left(i\right)}$$

In Formula (1), T is the average temperature per hour (°C), T_b_ is the lower critical growth temperature (°C), T_ob_ is the lower critical optimum temperature (°C), T_ou_ is the higher critical optimum temperature (°C), and T_m_ is the higher critical temperature (°C). During the fruit-ripening period, T_b_ = 15 °C, T_ob_ = 22 °C, T_ou_ = 28 °C, and T_m_ = 35 °C.

In Formula (2), HTEP is the hourly production of thermal effectiveness and PAR is MJ/(m^2^ h).

In Formula (3), DTEP is the daily production of thermal effectiveness and PAR is MJ/(m^2^ d).

In Formula (4), TEP_(i)_ is TEP after day i in MJ/m^2^ and TEP_(i+1)_ is TEP after i + 1 day in MJ/m^2^.

#### 2.2.2. Fruit Quality Parameters

In the second half of the year, tomatoes were collected at 5-day intervals from the time of color change until they were fully ripe (red) and 4 tomatoes of a uniform size and free from external pests and diseases were randomly selected at each time for the determination of fruit quality indicators. First, the color index was measured and then the fruit firmness was measured. Finally, the fruit was crushed with a sampler, quickly frozen with liquid nitrogen, and stored in an ultra-low-temperature refrigerator (−80 °C) to determine the biochemical indicators.

##### Appearance Quality Parameters

A digital fruit hardness tester (STEP Systems GmbH, Nuremberg, Germany) was used to measure the hardness of tomatoes. The color difference value was measured using a color difference meter (NR110; Shenzhen Tianyouli Standard Light Source Co., Ltd., Shenzhen, China). Three measurement points (evenly distributed) were selected in the tomato equatorial direction and the average value of the three measurement points was used as the CIE color space index of tomato fruit. L* represented black and white, a* represented red and green, and b* represented yellow and blue. It was then converted into a fruit color index (CI) [34].

(5) $$CI=2000\times a/\left({L^{*}({a^{*}}^{2}+{b^{*}}^{2})}^{0.5}\right)$$

##### Taste Quality Parameters

The soluble sugar content (SSC) was measured using the anthrone–sulfuric acid colorimetric method. Organic acid content (OAC) was titrated using a 0.1 M NaOH solution [43].

##### Nutrient Quality Parameters

Lycopene was measured using the spectrophotometric method. The vitamin C content (Vc) was measured using the 2, 6-dichloroindophenol titration method [43].

### 2.3. Comprehensive Evaluation Method of Tomato Fruit Quality

**Figure 1:**
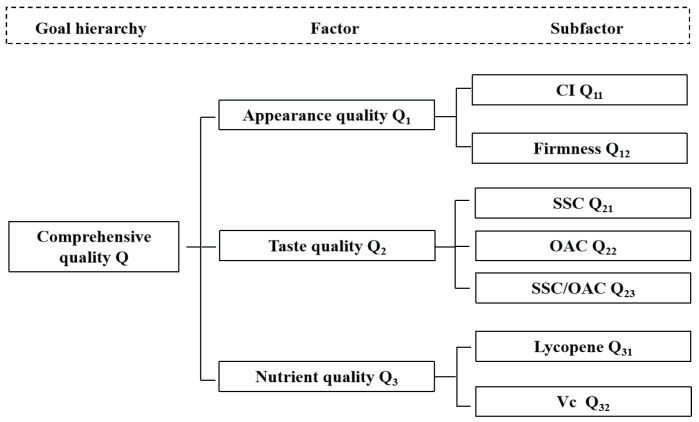
Evaluation hierarchy of comprehensive evaluation of tomato quality.

A hierarchical model for a comprehensive evaluation model of tomato fruit quality was established (Figure 1). All fruit quality indicators were divided into appearance quality, taste quality, and nutritional quality, which constituted the criterion layer. All secondary indicators were classified and recorded as sub-factors, which together constituted the scheme layer, including the fruit color index, firmness; SSC, OAC, SSC/OAC, lycopene, and Vc.

#### 2.3.1. Determination of Factor (Subjective) Weights via AHP

The analytic hierarchy process (AHP) is a technique and method that combines quantitative and qualitative methods to calculate decision weights to solve complex multi-factor problems. The method establishes a judgment matrix by measuring the relative importance of multiple factors, transforms the problem into the relative importance of comparative indicators, and determines the weight of each factor and sub-factor of the hierarchical model by quantitatively comparing each indicator. The indicator values in the judgment matrix are determined using a scale from 1 to 9 [40,44].

#### 2.3.2. Determination of Sub-Factor (Objective) Weights Using the Entropy Method

The entropy method is often used to determine the objective weight of an index. The greater the amount of information contained in the data, the smaller the entropy, indicating that the index has a greater impact on a comprehensive evaluation the higher the weight [36]. For the calculation of sub-factor weights using the entropy method, the measured data of the sub-factor set were initially standardized as follows:

(6) $$r_{ij}=\left(X_{ij}-min\left\{X_{ij}\right\}\right)/\left(max\left\{X_{ij}\right\}-min\left\{X_{ij}\right\}\right)\;(i=1,2,3,\ldots ,n;\;j=1,2,3,\ldots ,m)$$

where r_ij_ donates the standardized value. Subsequently, the specific gravity of these indices could be calculated as follows:

(7) $$P_{ij}=r_{ij}/\sum_{i=1}^{n}r_{ij}$$

The i-th factor and j sub-factor information entropy (e_j_) were defined as:

(8) $$e_{j}=-\frac{1}{\ln n}\sum_{i=1}^{n}p_{ij}\ln p_{ij}$$

Finally, the weight (w_ij_) of the j sub-factor was determined as follows:

(9) $$w_{ij}=\left(1-e_{j}\right)/\left(\sum_{j=1}^{m}\left(1-e_{j}\right)\right)$$

#### 2.3.3. Calculation of Combined Weights

Combined weights were obtained by multiplying the factor (subjective) weights obtained from AHP and the sub-factor (objective) weights obtained from the entropy method.

#### 2.3.4. Comprehensive Evaluation Based on TOPSIS

The TOPSIS method is a commonly used comprehensive evaluation method that can make full use of original data information to accurately reflect the advantages and disadvantages of each scheme.

After data standardization, a weighted decision matrix (Z) based on combination weights was established as follows:

(10) $$z=r_{ij}w_{ij}$$

where r_ij_ represents the measured data after standardization and w_ij_ represents the comprehensive weights based on game theory.

Then, the optimal and worst vectors constituted by the maximum and minimum values of each column of the weighted decision matrix were denoted as:

(11) $$Z^{+}=(Z_{max1},Z_{max2},\cdots Z_{maxn})$$

(12) $$Z^{-}=(Z_{min1},Z_{min2},\cdots Z_{minn})$$

where Z is the weighted decision matrix.

The distance between the weighted decision matrices Z^+^ and Z^−^ (D^+^ and D^−^) was calculated and, finally, the similarity of the i-th factor (C_j_) was calculated.

(13) $$D_{i}^{+}=\sqrt{\sum_{j=1}^{m}{\left(Z-Z_{j}^{+}\right)}^{2}}$$

(14) $$D_{i}^{-}=\sqrt{\sum_{j=1}^{m}{\left(Z-Z_{j}^{-}\right)}^{2}}$$

(15) $$C_{i}=D_{i}^{-}/\left(D_{i}^{+}+D_{i}^{-}\right)$$

### 2.4. Evaluation of Simulated Performance

In this study, the root mean square error (RMSE) was used to evaluate the reliability of the model. RMSE represents the relative error and absolute error between measured and simulated values, respectively [45]. The smaller the RMSE value, the higher the consistency between the simulated value and the measured value, indicating that a model can accurately and reliably predict results.

(16) $$RMSE=\sqrt{\frac{\sum_{i=1}^{n}{\left({SIM}_{i}-{OBS}_{i}\right)}^{2}}{n}}$$

where OBS_i_ represents the measured data, SIM_i_ represents the predicted data, and n refers to the number of samples.

### 2.5. Statistical Analysis

The experimental data were processed using Excel 2010. The weight of the entropy method was calculated using Python. The weight of AHP and the TOPSIS analysis were calculated using DPS.

## 3. Results

### 3.1. Environmental Data and Growth Characteristics of Tomato Quality

#### 3.1.1. Variations in Environmental Factors in the Solar Greenhouse throughout the Year

**Figure 2:**
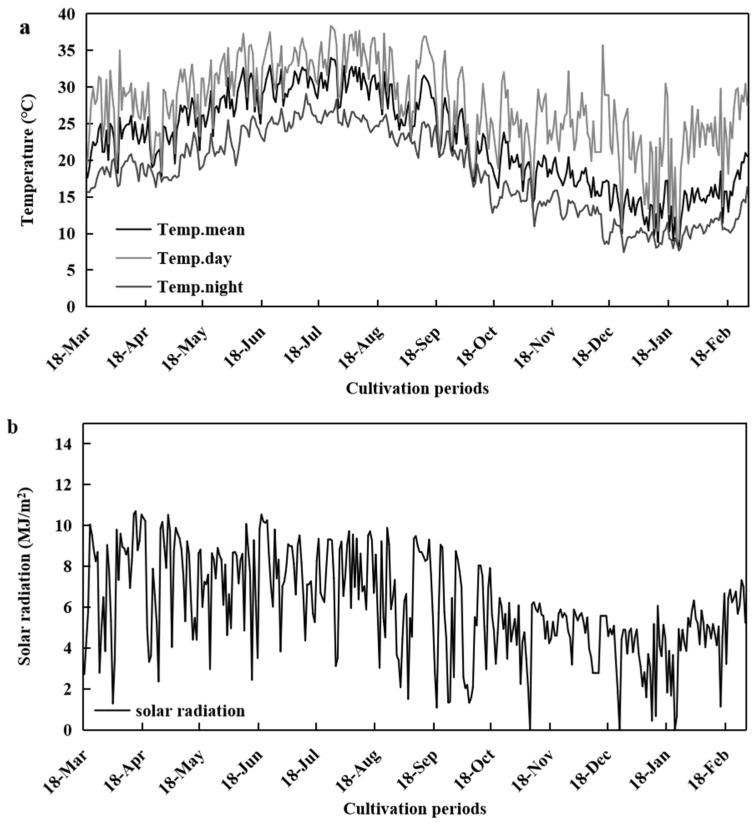
Variations in temperature (**a**) and solar radiation (**b**) in a solar greenhouse throughout the year. Temp.mean represents the average daily temperature, Temp.day represents the average daytime temperature, and Temp.night represents the average night-time temperature.

In order to understand the variations in temperature and light conditions in the solar greenhouse, we monitored environmental changes in real time from March 2021 to February 2022. As shown in Figure 2a, the daily mean air temperature increased from 17.62 °C in March 2021 to 33.99 °C in July and August 2021, then decreased to 10.75 °C in January 2022. The average temperature was above 18 °C in July and August, and the night temperature was above 15 °C from May to September. The durations of the daytime temperatures > 30 °C and night-time temperatures > 22 °C gradually increased, reaching a maximum in July before gradually decreasing. The longest durations of the most suitable daytime temperatures (18–30 °C) and night-time temperatures (15–22 °C) for tomato development were in April, September, and October, while the shortest were in July (Table S1).

As shown in Figure 2b, there were daily fluctuations in solar radiation throughout the tomato cultivation period. Significant solar radiation was recorded in the first half of the year, fluctuating around 9 MJ·m^2^·d^−1^; in the second half of the year, solar radiation decreased from August to the end of October, then remained stable (approximately 8 MJ·m^2^·d^−1^). This may have been caused by the shorter duration of daylight in winter and the covering of insulation at night. The average monthly sunshine hours increased and then decreased throughout the year, with June and July having the longest sunshine hours at 13.00 h and 13.45 h, respectively. January had the shortest average daylight hours at 7.65 h (Table S2).

#### 3.1.2. Tomato Fruit Quality at Different Planting Periods

In order to explore suitable temperature and light environment ranges for fruit quality formation during the ripening period, we planted 10 batches of tomatoes throughout the first and second half of the year in a solar greenhouse with natural environmental conditions (Table 1). The results showed that there was no significant difference in the color indices and SSC of tomatoes from different planting batches, while significant differences were found in OAC, Vc, lycopene, and fruit firmness (Table 2). As a single index cannot reflect the comprehensive quality of tomatoes, we conducted a comprehensive evaluation and analysis. Based on AHP and the entropy weight method, the comprehensive weights of the fruit quality indicators were obtained (Table 3). The TOPSIS method was used to obtain the comprehensive evaluation value of the tomatoes. The results showed that T6 had the highest comprehensive quality and the appropriate temperature and light ranges for the tomato fruit-ripening period were determined (Table 4).

### 3.2. Development of the Simulation Model for Tomato Quality

#### 3.2.1. Development of Tomato Quality Indices

In the first half of the year, at the red-ripening stage, there was no significant change in color values with the extension of the planting period (from T1 to T5). SSC, Vc, and lycopene slightly declined; OAC and fruit firmness slightly increased (Table 2). In the second half of the year, the tomato fruit quality indicators of five planting times were collected every 5 days from the completion of fruit expansion, including green-ripening stage (GM), veraison stage (V), and red-ripening stage (RR). The fruit quality indices are shown in Table 5. With a delay in planting, the duration tended to be longer in the veraison stage during fruit-ripening periods. a*, SSC, Vc, and lycopene significantly increased from mature green to red-ripening; L*, b*, OAC, and fruit firmness significantly declined. Therefore, the relationship between tomato quality and temperature and light was further investigated based on the data collected in the second half of the year.

We conducted a correlation analysis between the tomato quality and TEP, which was calculated using Equations (1)–(4). As shown in Table 6, there was a positive correlation between TEP and SSC/OAC (r = 0.917), SSC (r = 0.869), lycopene (r = 0.774), and Vc (r = 0.869). TEP was negative correlated with OAC (r = −0.897), and there were high and moderate correlations between TEP and fruit firmness (r = −0.823) and CI (r = 0.709). The effects of TEP on SSC, OAC, the sugar–acid ratio, and Vc were stronger than those of lycopene and fruit firmness. The SSC, OAC, and SSC/OAC of tomato fruit are the main components of taste quality and the main source of the tomato commodity value. Therefore, a tomato quality (SSC, OAC, and SSC/OAC) simulation model was constructed based on TEP to predict the effects of temperature and light on the tomato quality.

#### 3.2.2. Development of the Simulation Model for Tomato Fruit Single-Quality Indices

We established a simulation model based on TEP for the quality of tomatoes during fruit-ripening periods that included SSC, OAC, and SSC/OAC (Table 7). TEP was obtained using Formulas (1)–(4) to process the environmental data. The analysis showed that the relationship between SSC and TEP was consistent with the change in the logarithmic function curve and R^2^ was 0.750. The relationship between OAC and TEP conformed to the first-order function and R^2^ was 0.808. The sugar–acid ratio changed with TEP in the logarithmic function curve and R^2^ was 0.833.

**Figure 3:**
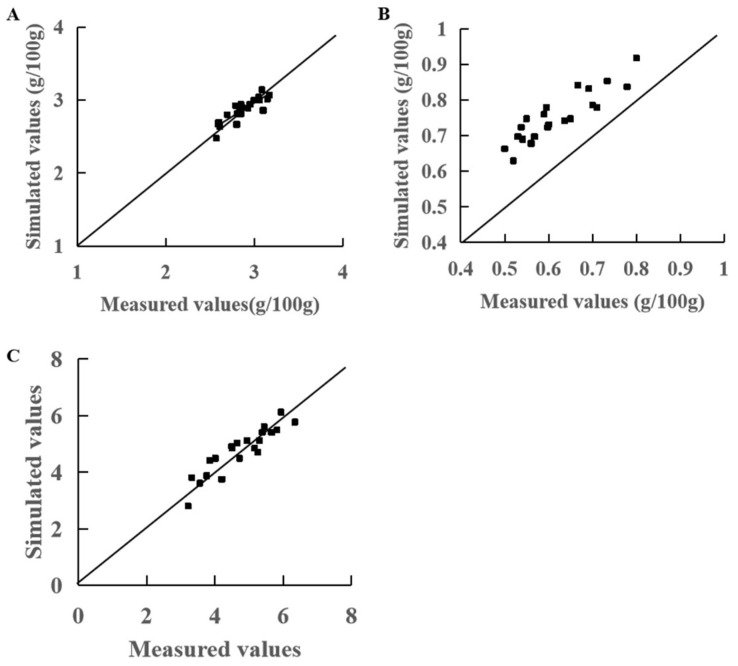
Comparison of simulated and measured values of tomato fruit quality. (**A**) SSC model validation. (**B**) OAC model validation. (**C**) SSC/OAC model validation.

In order to determine the accuracy of the model, the simulated values were calculated using the equation and compared with the measured values by calculating the RMSE. The results showed that the RSME value of the simulated models for SSC was 0.09%; this indicated that the model had high simulation accuracy and the variation trend of the simulated and measured values was the same (Figure 3A). The OAC simulation value had a large deviation from the measured value and the RMSE value was higher (0.14%), indicating that the model had moderate simulation accuracy (Figure 3B). The RSME value of the simulated models for the sugar–acid ratio was 0.358; this indicated that the model had high simulation accuracy (Figure 3C).

#### 3.2.3. Development of the Simulation Model for Tomato Comprehensive-Quality Indices

Tomato fruit quality is a comprehensive concept that is the result of the joint action of various quality indicators [33]. In order to evaluate tomato fruit quality using TOPSIS, the weight of factor (w_i_) in the first layer and the weight of sub-factor (w_ij_) in the second layer were calculated using AHP and the entropy method, respectively. These two sets of weights were then merged together (Table 8).

After obtaining the comprehensive weight of each quality index of tomato fruit, TOPSIS was used to calculate the comprehensive value of tomato fruit quality during the fruit-ripening periods (Table 9). The higher the comprehensive evaluation value, the higher the quality, indicating that the environmental parameters of the treatment were more conducive to obtaining high-quality tomatoes.

The simulation model was obtained by analyzing the relationship between the comprehensive evaluation value of fruit and TEP as follows (R^2^ = 0.757):

(17) $$y=1.189/\left(1+exp\left(-0.036(TEP-271.359)\right)\right)$$

where y is the comprehensive fruit quality evaluation value and 1.189, 0.036, and 271.359 are the parameters of the equation.

**Figure 4:**
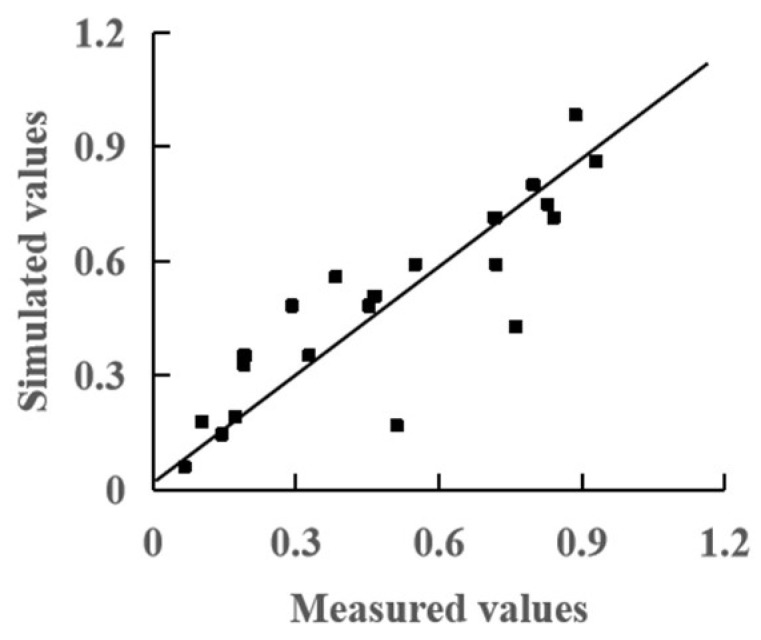
Comparison of simulated and measured comprehensive quality values of tomato fruit.

The RMSE value was 0.154, which showed that the simulation model provided medium precision (Figure 4).

## 4. Discussion

With the progress of tomato-planting technology, the yield of tomatoes has greatly improved. In recent years, high-quality tomatoes have gradually become the main target of market demand. In greenhouse cultivation, the environment is a key factor influencing the formation of tomato fruit quality and clarifying the relationship between environmental factors and the formation of quality is fundamental to achieve high-quality, high-yield tomatoes. Mathematical modeling has been used to describe the characteristics of crop growth. Establishing a growth model can help to better understand the responses of crops to their environment and improve the efficiency of agricultural production [46]. In this study, the relationship between fruit quality and environmental factors was studied.

A number of studies have shown that temperature and light stress can reduce quality formation in tomatoes, including high and low temperatures as well as strong and weak light [13,14,15,22]. However, a greenhouse environment is a multi-variable, highly coupled, and complex system. The regulation of environmental factors differs from the superposition of single factors [32]. There were significant differences in the quality of tomato fruit between the different planting periods (Table 2). This indicates that there is an association between environmental factors and tomato quality formation.

Previous studies have focused on analyzing the relationship between environmental factors and crop growth, and then have developed models to effectively predict crop growth, yield, and the duration of growth stage under different environmental conditions such as temperature and light, providing decision-making support for the environmental management of crop growth [28,30,47,48,49]. For example, a leaf area model was established based on the TEP method that uncovered the difference in plant growth caused by different light/dark cycle patterns from a physiological perspective [47]. However, there are fewer studies on the relationship between tomato fruit quality and environmental factors. In addition, previous crop simulation models mostly adopted single environmental factors such as GDD. However, GDD can only describe the effect of the lower growth-limit temperature and the upper growth-limit temperature on crop growth and development; it does not involve the effects of the photoperiod or high temperature on the retardation of development. Therefore, GDD is more suitable for field crops because the changes in field temperature and solar radiation are basically synchronous. In a cultivation facility, changes in environmental factors are not always synchronized due to heating, cooling, and the uncovering of insulation, so a simulation is less accurate when using a single environmental factor [50,51]. It is necessary to use a comprehensive index based on light and temperature to establish a simulation model [30,52]. The TEP method takes into account both air temperature and light, and can be used to build effective, simple models. In this study, based on the results of a correlation analysis, we established a simulation model for the fruit quality response based on TEP. The results showed that the SSC, OAC, and SSC/OAC of fruit quality in the fruit-ripening period were highly significantly correlated with TEP and the simulation accuracy was high (Table 7; Figure 3).

According to the model results of the different indicators we established (Table 5), it was found that the change trend of different quality indices was not consistent, indicating that it was difficult to achieve the optimal conditions of each index by adjusting the environmental factors. He et al. (2022) adopted a design of composite quadratic orthogonal regressive rotation with three factors and five levels, including temperature, relative humidity (RH), and photosynthetically active radiation (PAR), and established the response models of growth indicators (seedling index, dry-matter accumulation, net photosynthetic rate, transpiration rate, and chlorophyll content) for multiple environmental factors. The results showed that the most suitable environmental parameters of the five indicators were not consistent. It is clear that no single indicator can effectively reflect the growth of plants or the quality of fruit, so a comprehensive evaluation analysis is necessary. Here, the subjective and objective weights of all the quality indices were obtained based on AHP and the entropy method. Combining human subjective judgement and the objective information from index data can reflect the comprehensive quality of tomatoes during the fruit-ripening period in a more reasonable way [32,33,40]. This study comprehensively evaluated the fruit quality of tomatoes at different planting periods during the fruit-ripening period. The results showed that the optimal daily mean temperature during fruit ripening was 18.69 ± 1.35 °C, the daytime mean temperature was 26.30 ± 2.67 °C, the night-time mean temperature was 14.30 ± 1.21 °C, and PAR was 592.34 ± 88.74 μmol·m^−2^·s^−1^, with a sunshine duration of 9.86 ± 0.94 h·d^−1^ (Table 4). Then, we established a simulation model of comprehensive tomato fruit quality based on TEP using a regression analysis. The results showed that the comprehensive-quality formation model of tomato fruit at a mature stage based on TEP was moderately accurate (R^2^ = 0.757).

## 5. Conclusions

This study was carried out in the natural environmental conditions of a greenhouse. In total, 10 batches were grown within the range of environments in which tomatoes can be effectively grown, covering the environmental conditions that may be faced during the cultivation of tomatoes. Based on the correlation analysis between tomato fruit quality and environmental factors and the results of the integrated weighting of each quality index, the main quality indicators, including SSC, OAC, and SSC/OAC, were selected. A model was established to investigate the relationships between SSC, OAC, and SSC/OAC in tomato fruit and TEP during the fruit-ripening period in a solar greenhouse. The model was validated and the results indicated that the model had a high level of simulation accuracy. At the same time, based on a comprehensive evaluation analysis, a TEP-based comprehensive fruit quality formation model was established. The model was validated and the results indicated that the model had moderate simulation accuracy. This study provides decision-making support for the management of temperature and light in a heliostat during tomato ripening.

## Figures and Tables

**Table 1:** Dates of tomato planting.

	Planting Dates		Planting Date
T1	18 March 2021	T6	13 August 2021
T2	30 March 2021	T7	28 August 2021
T3	13 April 2021	T8	8 September 2021
T4	25 April 2021	T9	19 September 2021
T5	8 May 2021	T10	30 September 2021

**Table 2:** Tomato fruit quality at different planting periods.

	L*	a*	b*	CI	SSC (g/100 g)	OAC (g/100 g)	SSC/OAC	Vc (mg/100g)	Lycopene (mg/kg)	Fruit Firmness (kg/cm^2^)	Comprehensive Quality
T1	42.10 ± 2.00a	14.95 ± 2.61bc	13.69 ± 2.07abc	35.03 ± 2.94bc	3.26 ± 0.23a	0.52 ± 0.1cd	6.32	29.70 ± 1.21bc	21.57 ± 1.94ab	5.98 ± 1.51d	0.865
T2	41.13 ± 3.83ab	11.06 ± 1.33d	12.05 ± 3.61cd	32.87 ± 8.66c	3.19 ± 0.13ab	0.51 ± 0.02d	6.31	29.15 ± 0.90cd	19.97 ± 1.13bc	6.57 ± 1.05d	0.843
T3	41.68 ± 1.42a	16.27 ± 2.15abc	13.37 ± 1.85abc	37.08 ± 3.91abc	3.07 ± 0.12ab	0.60 ± 0.02b	5.12	26.75 ± 0.53d	19.60 ± 0.27c	7.98 ± 0.70bc	0.422
T4	39.24 ± 2.05c	14.21 ± 1.73c	11.28 ± 2.12d	39.93 ± 3.83a	3.10 ± 0.05ab	0.60 ± 0.04b	5.18	23.25 ± 1.91e	15.22 ± 0.29e	9.60 ± 2.30a	0.391
T5	41.67 ± 1.29a	14.91 ± 3.58bc	13.57 ± 2.56abc	35.50 ± 7.86bc	3.00 ± 0.04b	0.71 ± 0.04a	4.22	22.95 ± 1.91e	13.45 ± 2.15f	9.52 ± 1.01a	0.016
T6	41.30 ± 2.08ab	16.74 ± 2.06ab	13.99 ± 2.63abc	37.16 ± 4.85abc	3.17 ± 0.16ab	0.50 ± 0.03d	6.34	32.90 ± 0.76a	22.72 ± 0.65a	6.77 ± 1.04cd	0.895
T7	40.69 ± 1.64abc	17.67 ± 1.93a	14.27 ± 1.39ab	38.24 ± 2.66ab	3.10 ± 0.25ab	0.59 ± 0.02b	5.25	31.20 ± 4.07abc	22.37 ± 0.38a	8.03 ± 1.51bc	0.517
T8	40.72 ± 1.62abc	17.75 ± 2.64a	15.16 ± 2.63a	37.34 ± 2.83abc	3.09 ± 0.04ab	0.52 ± 0.01cd	5.94	32.60 ± 1.13ab	22.33 ± 1.81a	6.61 ± 0.33d	0.830
T9	39.58 ± 1.19bc	15.38 ± 2.46bc	12.91 ± 1.12bcd	38.71 ± 3.01ab	3.00 ± 0.22b	0.53 ± 0.02cd	5.66	32.25 ± 0.57ab	20.08 ± 1.17bc	7.35 ± 0.36bcd	0.749
T10	38.99 ± 1.15c	17.90 ± 1.21a	13.52 ± 1.29abc	40.93 ± 1.63a	3.15 ± 0.06ab	0.54 ± 0.03c	5.82	31.60 ± 1.62abc	17.79 ± 0.90d	8.33 ± 1.23ab	0.666

L* represents black and white, a* represents red and green, and b* represents yellow and blue. CI is color index, SSC is soluble sugar concentration, OAC is organic acid concentration, and Vc is vitamin C. Letters following the values of the indices of each season within rows are the significant differences according to Duncan’s multiple range tests at *p* < 0.05; presented values are means ± SD.

**Table 3:** Weight of tomato fruit quality at red-ripening stage.

Factor	w_Q1_		w_Q2_			w_Q3_	
Sub-Factor	w_Q11_	w_Q12_	w_Q21_	w_Q22_	w_Q23_	w_Q31_	w_Q32_
	0.158		0.680			0.162	
	0.457	0.543	0.320	0.519	0.161	0.426	0.574
Weight	0.072	0.086	0.218	0.353	0.109	0.069	0.093

**Table 4:** Optimum range of environmental factors at fruit-ripening stage.

Daily Mean Temperature (°C)	Daytime Mean Temperature (°C)	Night-Time Mean Temperature (°C)	PAR (μmol·m^−2^·s^−1^)	Insolation Duration (h·d^−1^)
18.69 ± 1.35	26.30 ± 2.67	14.30 ± 1.21	592.34 ± 88.74	9.86 ± 0.94

**Table 5:** Quality indices of tomatoes during fruit-ripening period and the corresponding TEP.

Treatments	Stages	T6	T7	T8	T9	T10
TEP	GM	249.70	232.49	248.64	245.40	260.16
	V_1_	267.65	247.68	258.81	260.16	266.53
	V_2_			269.87	266.53	271.29
	V_3_			280.41	271.29	276.58
	RR	283.39	263.97	290.10	276.58	278.11
L*	GM	47.02 ± 1.84bcd	48.83 ± 1.81ab	48.19 ± 1.20bc	46.69 ± 1.06cde	45.58 ± 1.85def
	V_1_	45.10 ± 3.58ef	44.38 ± 3.53fg	50.07 ± 2.22a	46.48 ± 2.62cde	47.58 ± 3.26bc
	V_2_			47.36 ± 1.98bcd	47.90 ± 1.29bc	43.78 ± 2.23fgh
	V_3_			42.19 ± 1.12hij	44.65 ± 1.71f	42.86 ± 1.95ghi
	RR	41.30 ± 2.08ijk	40.69 ± 1.64jkl	40.72 ± 1.62jkl	39.58 ± 1.19kl	38.99 ± 1.15l
a*	GM	−6.54 ± 0.62g	−6.95 ± 0.95g	−6.64 ± 0.43g	−6.49 ± 0.58g	−6.63 ± 0.64g
	V_1_	7.30 ± 5.05d	12.14 ± 3.30c	−6.98 ± 0.87g	−6.41 ± 1.12g	−5.17 ± 1.09g
	V_2_			−2.29 ± 1.14f	−0.09 ± 2.03e	7.84 ± 1.98d
	V_3_			17.73 ± 2.12a	11.77 ± 2.84c	16.58 ± 1.76ab
	RR	16.74 ± 2.06ab	17.67 ± 1.93a	17.75 ± 2.64a	15.38 ± 2.46b	17.90 ± 1.21a
b*	GM	22.51 ± 1.69abc	23.30 ± 1.79ab	23.19 ± 2.15ab	20.65 ± 2.39cde	20.35 ± 2.24de
	V_1_	19.23 ± 2.37e	16.34 ± 2.94f	23.73 ± 1.96a	20.73 ± 2.81cde	21.64 ± 2.44bcd
	V_2_			24.53 ± 3.41a	23.26 ± 3.22ab	15.38 ± 1.64fg
	V_3_			14.74 ± 1.61fgh	14.51 ± 1.80fgh	14.27 ± 1.95gh
	RR	13.99 ± 2.63gh	14.27 ± 1.39gh	15.16 ± 2.63fg	12.91 ± 1.12h	13.52 ± 1.29gh
CI	GM	−11.94 ± 1.75hi	−11.77 ± 0.92hi	−11.51 ± 8.48hi	−12.91 ± 3.38hi	−13.69 ± 4.85i
	V_1_	15.23 ± 1.80e	27.27 ± 1.62c	−11.29 ± 1.11hi	−12.93 ± 4.04hi	−9.84 ± 2.66h
	V_2_			−3.89 ± 2.80g	−0.40 ± 3.54f	20.49 ± 2.83d
	V_3_			36.34 ± 2.05b	27.87 ± 4.60c	35.42 ± 3.01b
	RR	37.16 ± 1.39b	38.24 ± 9.84ab	37.34 ± 1.78b	38.71 ± 3.35ab	40.93 ± 1.63a
SSC (g/100 g)	GM	2.60 ± 0.12f	2.57 ± 0.24f	2.59 ± 0.12f	2.61 ± 0.10ef	2.85 ± 0.26bcdef
	V_1_	2.86 ± 0.18abcde	2.80 ± 0.03bcdef	2.69 ± 0.12def	2.81 ± 0.20bcdef	2.93 ± 0.14abcd
	V_2_			2.79 ± 0.16cdef	2.84 ± 0.22bcdef	2.95 ± 0.18abcd
	V_3_			3.05 ± 0.09abc	2.85 ± 0.11bcdef	3.06 ± 0.34abc
	RR	3.17 ± 0.16a	3.10 ± 0.25ab	3.09 ± 0.04abc	3.00 ± 0.22abc	3.15 ± 0.06a
OAC (g/100 g)	GM	0.69 ± 0.05cde	0.80 ± 0.01a	0.78 ± 0.03ab	0.73 ± 0.13bc	0.71 ± 0.02cd
	V_1_	0.64 ± 0.03efg	0.67 ± 0.04de	0.70 ± 0.02cd	0.59 ± 0.03fghi	0.65 ± 0.01def
	V_2_			0.60 ± 0.03fgh	0.55 ± 0.01hijk	0.60 ± 0.03fgh
	V_3_			0.56 ± 0.02hij	0.54 ± 0.01ijk	0.57 ± 0.03hij
	RR	0.50 ± 0.03k	0.59 ± 0.02ghi	0.52 ± 0.01jk	0.53 ± 0.02jk	0.54 ± 0.03hijk
SSC/OAC	GM	3.76	3.21	3.32	3.56	4.01
	V_1_	4.49	4.20	3.85	4.72	4.51
	V_2_			4.65	5.16	4.93
	V_3_			5.44	5.30	5.39
	RR	6.34	5.25	5.94	5.66	5.82
Vc (mg/100 g)	GM	16.15 ± 0.53e	16.95 ± 0.50e	16.85 ± 0.53e	15.30 ± 1.64e	17.35 ± 0.90e
	V_1_	26.25 ± 0.30bc	24.00 ± 1.23c	20.85 ± 3.92d	20.05 ± 0.62d	26.10 ± 0.82bc
	V_2_			25.90 ± 0.77bc	28.30 ± 2.61b	28.15 ± 1.62b
	V_3_			31.65 ± 0.25a	31.65 ± 0.82a	31.60 ± 1.40a
	RR	32.90 ± 0.76a	31.20 ± 4.07a	32.60 ± 1.13a	32.25 ± 0.57a	31.60 ± 1.62a
Lycopene (mg/kg)	GM	4.40 ± 0.22i	4.18 ± 0.22i	6.75 ± 0.46h	7.32 ± 0.13gh	6.84 ± 0.53h
	V_1_	9.90 ± 2.91ef	11.44 ± 0.90e	6.93 ± 0.58h	7.23 ± 0.11gh	7.93 ± 0.52gh
	V_2_			10.90 ± 0.56e	8.96 ± 0.81fg	10.26 ± 0.32ef
	V_3_			21.89 ± 3.80a	16.37 ± 0.26c	13.78 ± 0.58d
	RR	22.72 ± 0.65a	22.37 ± 0.38a	22.32 ± 1.81a	20.08 ± 1.17b	17.79 ± 0.90c
Fruit firmness (kg/cm^2^)	GM	13.53 ± 1.50bc	15.39 ± 0.92a	15.03 ± 0.27a	14.99 ± 0.53a	14.64 ± 0.77ab
	V_1_	10.18 ± 1.12e	11.57 ± 0.90d	13.68 ± 0.49bc	13.53 ± 0.59bc	13.57 ± 1.05bc
	V_2_			8.76 ± 1.14fg	13.10 ± 0.59c	12.46 ± 1.47cd
	V_3_			7.45 ± 0.98hi	8.78 ± 1.08fg	9.82 ± 2.83ef
	RR	6.77 ± 1.04j	8.03 ± 1.51gh	6.60 ± 0.33ij	7.35 ± 0.36hi	8.33 ± 1.23gh

Notes: GM, V, and RR represent the green mature stage, the veraison stage, and the red-ripening stage, respectively. Different letters represent significant differences at *p* < 0.05 level.

**Table 6:** Correlation coefficients (r) for tomato fruit quality and TEP.

	CI	SSC	OAC	SSC/OAC	Lycopene	Vc	Fruit Firmness
TEP	0.709 **	0.869 **	−0.897 **	0.917 **	0.774 **	0.869 **	−0.823 **

Notes: ** indicates significant levels at *p* < 0.01.

**Table 7:** Simulation model of tomato fruit quality index based on TEP.

Fruit Quality	Model Equations	a	b	R^2^	RMSE
SSC	SSC=aln⁡X+b	0.031	0.139	0.750	0.09%
OAC	OAC=aX+b	−5 × 10^−5^	0.021	0.808	0.14%
SSC/OAC	y=aln⁡X+b	14.989	−78.874	0.833	0.358

Note: X is TEP (MJ/m^2^); a and b are parameters of the equation. RMSE is the root mean square error.

**Table 8:** Weight of tomato fruit quality during the fruit-ripening periods.

Factor	w_Q1_		w_Q2_			w_Q3_	
Sub-Factor	w_Q11_	w_Q12_	w_Q21_	w_Q22_	w_Q23_	w_Q31_	w_Q32_
	0.158		0.680			0.162	
	0.628	0.372	0.335	0.374	0.290	0.567	0.432
Weight	0.099	0.059	0.228	0.254	0.197	0.092	0.070

**Table 9:** The tomato comprehensive-quality indices based on TOPSIS.

	The Green Mature Stage	The Veraison Stage			The Red-Ripening Stage
T6	0.120	0.458			0.953
T7	0.028	0.531			0.799
T8	0.065	0.145	0.346	0.831	0.920
T9	0.098	0.275	0.409	0.720	0.870
T10	0.151	0.254	0.546	0.733	0.852

## Data Availability

Data are contained within the article and Supplementary Materials.

## Supplementary Materials

The following supporting information can be downloaded at: https://www.mdpi.com/article/10.3390/plants13121662/s1.
