# Supplementary material for: Testing a Simulation Model for the Response of Tomato Fruit Quality Formation to Temperature and Light in Solar Greenhouses
Source: Plants (Basel). 2024 Jun 15;13(12):1662. doi: 10.3390/plants13121662 (PMC11207517; doi:10.3390/plants13121662)
Supplement: Supplementary file 1 [file plants-13-01662-s001.zip › plants-3006209-supplementary.pdf]

Supplementary data

Table S1. Temperature and light analysis of solar greenhouse in different months.

Date (year-month)	Duration of day temperature（h·day $^{-1}$）				Duration of night temperature（h·night $^{-1}$）			
	<12 ℃ (h)	12-18 ℃ (h)	18-30 ℃ (h)	≥30 ℃ (h)	<10℃(h)	10-15℃(h)	15-22 ℃ (h)	>22 ℃ (h)
2021.03	0.94	1.19	3.85	3.74	2.90	4.23	6.76	0.99
2021.04	0.00	1.23	6.60	3.40	0.00	0.63	9.80	0.73
2021.05	0.00	0.61	5.29	6.30	0.00	0.13	6.52	5.16
2021.06	0.00	0.10	5.00	7.80	0.00	0.00	2.00	9.00
2021.07	0.00	0.00	3.45	10.00	0.00	0.00	0.00	10.55
2021.08	0.00	0.00	4.39	8.35	0.00	0.00	0.84	10.42
2021.09	0.00	0.07	6.07	5.30	0.00	0.00	5.60	6.97
2021.10	0.06	1.45	5.74	3.00	0.13	3.39	9.42	0.81
2021.11	0.10	1.63	4.60	2.43	0.37	9.10	5.73	0.03
2021.12	0.61	1.10	4.06	2.00	5.55	9.32	1.35	0.00
2022.01	1.90	1.42	3.32	1.00	10.61	5.26	0.48	0.00
2022.02	0.50	1.04	4.89	1.82	5.82	7.39	2.54	0.00

Table S2. Analysis of sunshine hours in different months in solar greenhouse.

Date (year-month)	Insolation hours(h)	<200 μmol·m $^{-2}$·s $^{-1}$ (h)	200-500 μmol·m $^{-2}$·s $^{-1}$ (h)	500-1200 μmol· m $^{-2}$·s $^{-1}$ (h)	≥1200 μmol·m $^{-2}$·s $^{-1}$ (h)
2021.03	9.72	0.76	1.73	5.68	1.55
2021.04	11.23	0.93	1.97	3.63	4.70
2021.05	12.88	1.23	1.81	4.39	4.77
2021.06	13.00	1.00	2.50	5.37	4.13
2021.07	13.45	1.00	2.68	5.61	4.16
2021.08	12.74	0.90	2.71	5.55	3.58
2021.09	11.43	1.10	2.53	4.83	2.97
2021.10	10.26	0.84	2.71	5.61	1.10
2021.11	8.77	0.50	1.20	6.70	0.37
2021.12	7.77	0.19	0.84	6.71	0.03
2022.01	7.65	0.35	1.65	5.42	0.23
2022.02	8.25	0.04	0.79	5.79	1.64
